# Pathophysiology and Treatment Opportunities of Iron Deficiency in Heart Failure: Is There a Need for Further Trials?

**DOI:** 10.1007/s11897-023-00611-3

**Published:** 2023-07-10

**Authors:** Michał Tkaczyszyn, Marat Fudim, Piotr Ponikowski, Jan Biegus

**Affiliations:** 1 Institute of Heart Diseases, Wroclaw Medical University, Borowska 213, 50-556 Wroclaw, Poland; 2 Institute of Heart Diseases, University Hospital, Wroclaw, Poland; 3 Division of Cardiology, Duke University Medical Center, Durham, NC USA; 4 Duke Clinical Research Institute, Durham, NC USA

**Keywords:** Heart failure, Iron deficiency, Ferric carboxymaltose, Intravenous iron

## Abstract

**Purpose of Review:**

Iron deficiency (ID) complicates heart failure (HF) at different stages of the natural history of the disease; however, this frequent comorbidity is still not comprehensively understood and investigated in terms of pathophysiology. Intravenous iron therapy with ferric carboxymaltose (FCM) should be considered to improve the quality of life, exercise capacity, and symptoms in stable HF with ID, as well as to reduce HF hospitalizations in iron-deficient patients stabilized after an episode of acute HF. The therapy with intravenous iron, however, continues to generate important clinical questions for cardiologists.

**Recent Findings:**

In the current paper, we discuss the class effect concept for intravenous iron formulations beyond FCM, based on the experiences of nephrologists who administer different intravenous iron formulations in advanced chronic kidney disease complicated with ID and anemia. Furthermore, we discuss the neutral effects of oral iron therapy in patients with HF, because there are still some reasons to further explore this route of supplementation. The different definitions of ID applied in HF studies and new doubts regarding possible interactions of intravenous iron with sodium-glucose co-transporter type 2 inhibitors are also emphasized.

**Summary:**

The experiences of other medical specializations may provide new information on how to optimally replenish iron in patients with HF and ID.

## Introduction

**Fig. 1:**
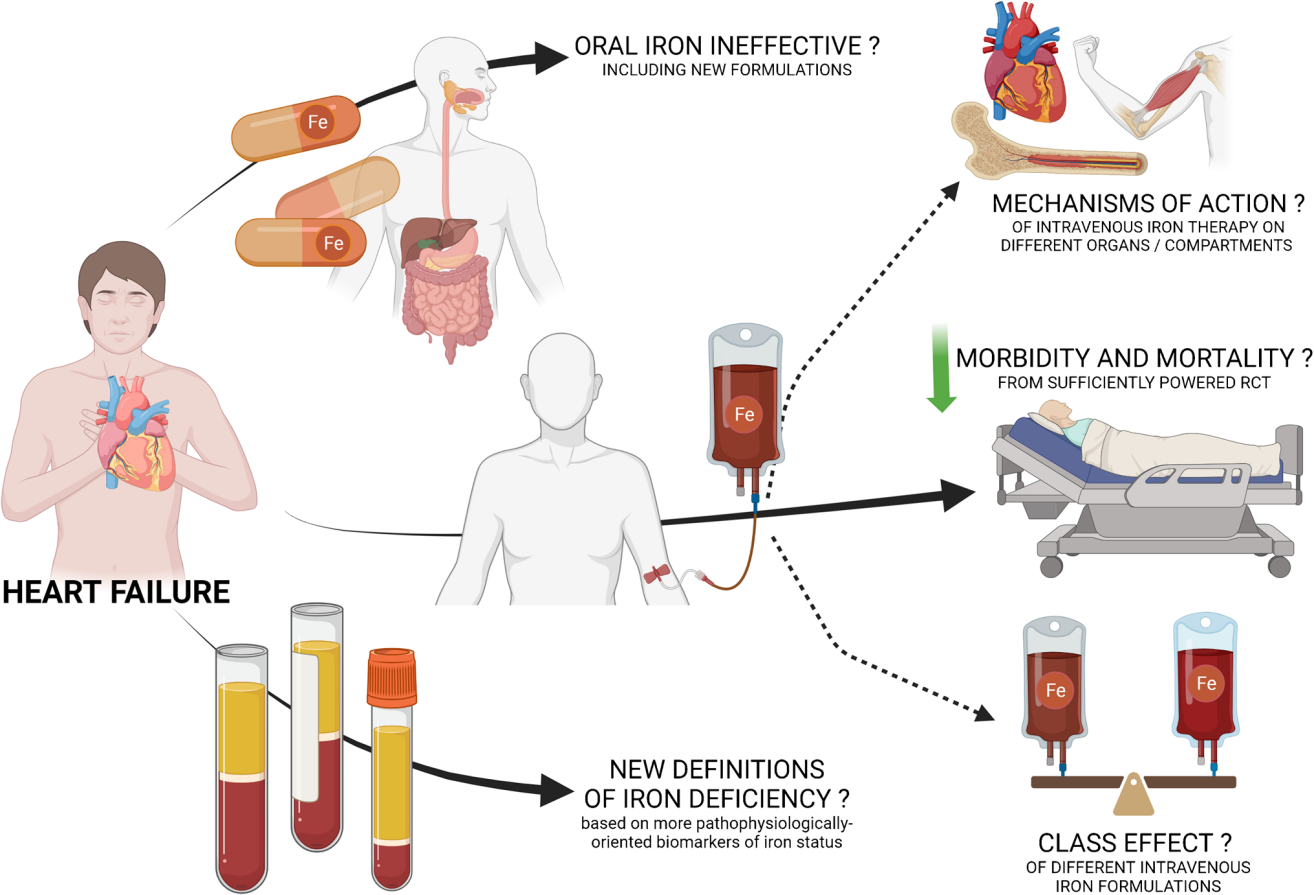
Key clinically relevant questions regarding iron therapy in heart failure patients. Created with BioRender.com. *RCT* randomized clinical trial

Iron deficiency (ID) complicates heart failure (HF) at different stages of the natural history of the disease. Although ID is still not comprehensively understood and explored in terms of exact pathophysiology, it has been demonstrated to indisputably worsen symptoms, quality of life (QoL), and clinical outcomes (including re-hospitalizations) in HF patients, and these detrimental effects are largely independent of anemia [1, 2, 3••]. Moreover, uniformly defined ID complicating stable or recently acutely decompensated HF is evidence-based amenable for effective and safe parenteral pharmacological correction. Current HF guidelines clearly recommend to screen HF patients for ID irrespective of actual clinical trajectory of the disease, and to consider the replenishment of this micronutrient using ferric carboxymaltose (FCM—one of i.v. iron formulations) to improve symptoms, QoL, exercise capacity, and if such deficiency is detected in clinically stabilized acute HF patients pre-discharge, also re-hospitalizations [4]. Importantly, there is still no conclusive data on the prognostic benefit (morbidity and mortality) of intravenous iron therapy in stable/chronic patient population because initial trials in this field were focused on QoL, symptoms, and exercise capacity of patients [5, 6]. Clear evidence regarding “hard” clinical outcomes, if positive, would position disordered iron status as one of the key additional therapeutic targets after co-initiating foundational chronic HF drugs, taking into account the prevalence of ID as well as ease and cost-effectiveness of its correction (see below). Moreover, two further large randomized clinical trials (RCTs) designed to investigate prognostic benefits of i.v. iron (HF hospitalization and cardiovascular death as a combined primary outcome) that recruited patients hospitalized for HF (in the AFFIRM-AHF trial, all recruited HF patients were stabilized after acute HF, whereas in an IRONMAN trial, one-third of study participants were currently or recently hospitalized for HF) coincided with the outbreak of the COVID-19 pandemic, which makes it necessary to approach the neural results (lack of superiority) with some caution [7, 8]. We also have new additional analyses available—for example, in a recent Bayesian meta-analysis combining individual patient data from FAIR-HF, CONFIRM-HF, and AFFIRM-AHF trials with IRONMAN results (a total of more than 3 thousands of patients were included in the analysis), it has been demonstrated that in a broad range of HF with reduced ejection fraction (HFrEF) patients with ID the treatment with intravenous iron reduced the composite of HF hospitalization or cardiovascular death [9••]. Therefore, the results of still recruiting phase 4 study FAIR-HF2 (NCT03036462) are particularly awaited (planned completion date: 2024). The study will be the first sufficiently powered prospective, multicenter, double-blind randomized controlled trial (RCT) to determine if intravenous FCM administered in iron-deficient stable HFrEF patients (hospitalized and re-stabilized or stable ambulatory patients with either a history of HF hospitalization or currently significantly elevated natriuretic peptides; all with left ventricular ejection fraction (LVEF) <45% and in a broad range of hemoglobin [Hb] status) compared to placebo can reduce combined rate of recurrent hospitalizations for HF and of cardiovascular death. Another active (NCT03037931) double-blind, multicenter, prospective, RCT to investigate the effects of FCM versus placebo on the all-cause mortality within 12 months, 12-month hospitalization for HF, and 6-month exercise capacity (primary outcomes) in patients with stable HFrEF and ID is HEART-FID trial, but the status of the study is “not recruiting” at ClinicalTrials.gov (access date: 03 Apr 2023). Nevertheless, intravenous iron therapy has sanctioned guideline-based indications for administration to improve the above-mentioned QoL and physical capacity/symptomatology in stable HF as well as re-hospitalizations in acute HF patients, which therapy continues to generate some clinical questions for physicians-practitioners (Fig. 1). In the current paper, we discuss the class effect concept for intravenous iron formulations as well as neutral effects of oral iron therapy in patients with HF. Furthermore, the different definitions of ID applied in HF studies and new doubts regarding possible interactions of intravenous iron with sodium-glucose co-transporter type 2 inhibitors are also highlighted.

## Beyond Ferric Carboxymaltose—Is There a Need to Check Other I.V. Iron Formulations?

As FCM has been used almost exclusively in large double-blinded RCTs on intravenous iron supplementation in HF patients with concomitant ID, the current ESC guidelines [4] mention the therapy with this formulation only to be considered (level of evidence IIa) in patients either in the chronic phase of the disease or pre-discharge after HF exacerbation (acute HF hospitalization). Only a few smaller RCTs investigated another iron preparation (iron sucrose) (e.g.,) [8, 10, 11], and thus, in the context of HF, only FCM should be considered. On the other hand, we have some data suggesting that other i.v. iron preparations may also be effective in the context of some clinical endpoints. In a recently published investigator-initiated, prospective, randomized, open-label, blinded-endpoint, multicenter (UK-based) IRONMAN trial, more than 1100 adult patients with HFrEF (LVEF ≤ 45%) and ID (transferrin saturation [TSAT] < 0.2 or serum ferritin < 100 μg/L) were randomized 1:1 to receive either intravenous ferric derisomaltose (formerly called iron isomaltoside) or usual care and followed-up for a median of 2.7 years, and the dosing was based as usual in iron studies on patient body mass and Hb [8, 12]. Although the primary endpoint of the trial (recurrent hospitalizations for HF and cardiovascular death) fell short of conventional statistical significance (22.4 vs. 27.5 per 100 patient-years in i.v. iron vs control group, respectively; rate ratio [RR] 0.82, 95% confidence interval [CI] 0.66–1.02, *p* = 0.07), in the pre-specified COVID-19 analysis, there were fewer primary endpoints in the ferric derisomaltose group compared with controls (22.3 vs. 29.3/100 patient-years; RR 0.76, 95% CI 0.58–1.00; *p* = 0.047) [8]. The question about the potential class effect of i.v. iron preparations for the therapy of ID in HF seems even more justified, because although FCM has been proven to be cost-effective in economic evaluation analyses [13–15], it is certainly not available in all hospitals that commonly take care for patients with HF, as it is one of the most common causes of unplanned re-hospitalizations in elderly patients [16]. This problem has already been discussed in the field of nephrology, in the context of replenishing iron using its different intravenous formulations in patients with moderate to severe renal insufficiency, who are frequently anemic, and ID is one of the crucial contributors to decreased hemoglobin concentration. It needs to be acknowledged that a few currently available in European Union intravenous iron formulations share common colloidal structure of central elemental iron deposit (iron oxy-hydroxide core) surrounded by a carbohydrate shell preventing from its uncontrolled rapid release [2, 17, 18]. Despite a common general chemical configuration, it is the differences in the size of the core and those regarding the structure of the shell that ultimately cause individual preparations to differ in bioavailability, pharmacokinetics, allowed dosage (frequency and maximal single loading dose), and—to some extent—also side effects [17–19]. Nevertheless, it should be emphasized that the available data (mainly from the nephrology and hematology literature) indicate that there are no significant clinical differences in terms of efficacy and safety between the individual intravenous iron preparations currently available on the market, and in everyday clinical practice—regarding patients with renal insufficiency—when choosing a specific formulation, the local availability as well as patient convenience are primarily taken into account (e.g., if patient does not require regular contact with healthcare, for example, because of hemodialysis program, it is more convenient to administer higher doses of iron but less often) [20, 21]. It is noted, however, that many trials investigating i.v. iron  analyzed anemia and/or iron parameters as surrogates of therapeutic efficacy [20], and therefore, there are still needed large RCTs targeting “hard” clinical endpoints (in different patient populations), the inclusion and detailed analysis of which is a great advantage of the research on i.v. iron in HF patients. In conclusion, no recommendation can be made at this stage for any other intravenous iron preparation other than FCM, although other formulations may be the subject of RCT research for efficacy and safety in the future, which has some theoretical (similar structure of different intravenous iron preparations) and practical premises from other fields of medicine (nephrology and hematology).

## Further Testing Oral Iron—Kicking Closed Doors?

Another issue bothering cardiologists managing HF patients concerns the complete omission of oral iron therapy in the recommendations as an ineffective form of supplementation in the setting of chronic disorders. Is the topic actually closed and further research unjustified? Although newer oral iron preparations are constantly being developed (better bioavailability and fewer gastrointestinal side effects are at stake), the results of one neutral RCT have led to the conclusion that oral iron does not bring clinical benefits in HF patients [22, 23]. In a double-blind RCT IRONOUT [23], more than 200 of patients with stable, symptomatic (New York Heart Association class II, 67%; others, III) HFrEF (LVEF ≤ 40%) and with concomitant ID (defined as serum ferritin 15–100 ng/mL or 100–299 ng/mL with TSAT < 20%) were randomly assigned to receive oral iron polysaccharide (150 mg bid—several times more than the recommended daily dose of iron to overcome possible poor absorption) or placebo for 16 weeks, and the primary endpoint analyzed was a change in cardiopulmonary exercise testing peak oxygen consumption from baseline to week 16. Patients receiving iron vs placebo did not differ regarding the primary outcome measure, nor there were differences in 6-min walking test distance, neurohormonal activation or health-related quality of life in iron-replete v.s. controls from baseline up to week 16 [23]. The repletion efficacy itself requires additional comment, as the investigators unequivocally evaluated that oral iron “minimally repleted iron stores,” which is reflected in only a slight improvement of indices of iron status (distinctly TSAT and borderline serum ferritin) after 16 weeks of therapy [23]. The mechanistic question should be recalled again here—why iron p.o. hardly works in HF? The potential pathophysiology of this phenomenon should be sought primarily at the level of the gastrointestinal tract, where several local and systemic mechanisms contribute to poor absorption of iron regardless of the supply in patients with chronic diseases [24, 25]. It needs to be acknowledged that the functioning of gastrointestinal tract is impaired in the course of HF and overlapping intestinal congestion, hypoperfusion and abnormalities regarding gut microbiota favor malabsorption processes and malnutrition [26, 27]. Indeed, significant proportion of HF patients presents with the deficiency in one or more micronutrients [28–30]; however, it is worth noting that—in general—apart from intravenous iron, there is no supporting evidence to supplement/administer any other microelement in such patients [4]. Not only direct (local) gastrointestinal pathologies but also the “environment” of systemic low-grade inflammation is considered to contribute to decreased iron absorption within intestines and its abnormal distribution within particular tissue compartments [25, 31]. A special role here is attributed to the acute-phase antimicrobial peptide hepcidin, which is one of the main regulators of iron absorption at the intestinal level and not only-hepcidin oversees this key “checkpoint,” because the organism cannot actively remove iron, so it has to dynamically block transferring iron to circulation within gastrointestinal system (but also from, e.g., mononuclear phagocyte system [MPS]) in the situation of sufficient supply of the body with this micronutrient [19, 32]. However, in the course of chronic inflammation, the overexpression of hepcidin inhibits the transportation of iron to the circulation not only from the enterocyte but also MPS and liver, which contributes to the deficiency of this nutrient through the so-called maldistribution [22, 25, 31]. This is an important contributing mechanism of the anemia of chronic disease such as chronic kidney disease (CKD) [32], but, on the other hand, we have some evidence that the hepcidin axis may not be altered in the same way in HF as in other chronic diseases. For example, it has been demonstrated in one observational study in HFrEF patients that although circulating hepcidin predicted worse prognosis in study participants, the highest levels of this peptide were measured in less advanced stages of the disease (when ID is yet less prevalent) and neither greater hepcidin correlated with higher pro-inflammatory biomarkers (CRP, IL-6), nor it was clearly related to anemia status [33]. It needs to be highlighted that the complex mechanisms of anemia and/or ID in CKD and HF do not completely overlap (e.g., failing kidneys do not produce sufficient amounts of endogenous erythropoietin and this is the reason for exogenous erythropoiesis-stimulating agents supplementation with “conditioning” using iron formulations in this group of patients), but systemic pro-inflammatory activation is a common element for these two diseases [22]. Indeed, nephrologists also face the problem of insufficient absorption of oral iron (e.g. older, conventional formulations such as ferrous sulfate), but there are a number of new achievements in this area comprehensively reviewed elsewhere—namely, there is limited but promising evidence that newer formulations such as ferric citrate, ferric maltol, or sucrosomial iron, are able to improve iron and erythropoietic parameters in the clinical setting of advanced CKD, including patients on dialysis [22]. In general, when referring to the evidence-based, high-quality data on the superiority of intravenous vs. oral iron in CKD patients, the quality of evidence showing the superiority of the intravenous form is not as strong as clinicians would expect. O’Lone et al. analyzed the results of 39 studies (yielding a total number of more than 3800 adults and children) comparing intravenous and oral iron therapy in CKD (also undergoing renal replacement therapy with dialysis), and have concluded that although i.v. iron increases iron indices and hemoglobin more effectively than oral, the evidence on the impact on other significant outcomes such as all-cause and cardiovascular death or even quality of life was uncertain [34]. Regarding newer oral iron formulations, we also have some data for HF suggesting that its oral form may not be ruled out entirely. In one small, not randomized, open-label study that recruited 50 patients with HFrEF and concomitant ID, it has been demonstrated that oral sucrosomial iron administered for 3 months improved hemoglobin concentration, serum iron, and serum ferritin in all treated study participants [35]. Although the additional analyzed data on the improvement of physical capacity from this study—as results particularly sensitive to its open-label design—should be interpreted with caution, it should be emphasized that sucrosomial iron was in general well tolerated and only one patient discontinued active treatment due to drug-related gastrointestinal symptoms [35].

## Iron Deficiency and the Heart—Uncertainties Regarding Definitions, Mechanisms, and Safety

Although considered to have some disadvantages, the definition of ID commonly used in HF patients has one absolutely key advantage (unlike many other definitions in internal medicine)—it has been proven in large RCTs to discriminate patients with HF who benefit from intravenous FCM administration [3••]. Although there have been concepts and proposals of more pathophysiologically-oriented definitions, including those taking into account more appropriate, but less available, indices of comprehensive iron status from peripheral blood, each of them has to face the fact of the lack of RCTs using such a methodology (Table 1). On the other hand, it seems the definition of ID valid for stable or acute HF patients should not be extrapolated too easily to other populations of patients with cardiovascular diseases—for example, only recently it has been demonstrated for patients with pulmonary vascular disease that, firstly, the definition of ID analogous to HF does not discriminate well between patients in terms of exercise capacity and functional status, and secondly, the definition taking into account only decreased TSAT (with a slightly changed threshold, i.e., < 21%) adequately predicts worse functional status, cardiac remodeling, and outcomes [36].

**Table 1:** Brief summary of different approaches on how to define and diagnose iron deficiency (ID) in heart failure (HF) patients that have been applied in observational and interventional studies

Definition/approach how to define/diagnose ID in HF	Advantages	Disadvantages
Serum ferritin < 100 µg/L or 100–299 µg/L when TSAT < 20% (there were many minor modifications of this definition, e.g., different cut-offs or even omission of ferritin)	- Original definition verified in large RCTs on intravenous FCM (chronological: FAIR-HF, CONFIRM-HF, EFFECT-HF, AFFIRM-AHF, IRONMAN)	- Arbitrary cut-offs - Sensitive to inflammation - Too easily extended to other populations, e.g., PH
Serum iron < LLN	- Easily accessible	- Without taking into account the complex iron homeostasis in the body - Not verified in large RCTs
Decreased hepcidin + elevated sTfR (healthy population percentiles-based cut-offs)	- Strongly valid pathophysiologically	- Not verified in large RCTs - Not widely accessible
Bone marrow iron depletion	- Sensitive and specific assessment - Direct evaluation of ID	- Invasive, demanding assessment, and therefore virtually inaccessible - Not verified in RCTs
Myocardial iron depletion	- Valid pathophysiologically (?)	- Emerging but hypothetical approach only - Uncertain significance, also in terms of potential pharmacological correction - Lack of standardization (indirect measurement vs direct tissue analysis?) - Not verified in large RCTs

*TSAT* transferrin saturation, *sTfR* soluble transferrin receptor, *PH* pulmonary hypertension, *LLN* lower limit of normal, *FCM* ferric carboxymaltose

It needs to be acknowledged, that we still do not know exactly how FCM administered to patients with HFrEF and systemic ID (measured based on peripheral blood analysis) brings multifaceted clinical benefits. Improving the functioning of the bone marrow and the erythroid line cannot be a key element, as iron works across a wide spectrum of Hb and red cell indices, and even in patients without anemia [25, 37]. Effects on myocardial energetics are difficult to prove, although we have some limited mechanistic data on improving skeletal muscle energetics [38••], as well as a few proof-of-concept experiments in rodents and cell lines reviewed in detail elsewhere, demonstrating that abnormal cardiomyocyte energetics due to induced less or more severe ID (and also consequent anemia) can be reversed through the rapid replenishment of the missing iron [39, 40]. It remains also unknown why, for example, in non-ischemic HF FCM does not impact “hard” clinical outcomes as it does in patients with primary ischemic etiology of the disease, as demonstrated in one of the sub-analyses of the AFFIRM-AHF trial [41]. Hypothetical concepts of the not always beneficial effects of FCM on the compromised heart and kidneys through circulating regulators of calcium and phosphate metabolism have been proposed and commented, which indisputably requires further research [42]. There is also some anecdotal but intriguing evidence that FCM affects specific iron compartments in the body, including the heart—Gertler et al. [43] tracked indirectly iron signal using cardiac magnetic resonance–based approach in chronic HF patients treated with FCM for ID and have detected its increase within left ventricle, spleen, and liver after such therapy. Importantly, the assumptions formulated in the public space that an intensive loading of intravenous iron may be harmful in HF by, for example, inducing rapid oxidative stress in dedicated tissues [44] have not been confirmed because, firstly, there is no excess of adverse events in available RCTs. Secondly, iron works even in acute HF, and this is an a priori population with a highly dysregulated metabolism at tissue and cells in different organs including liver and kidneys, despite the achievement of relative clinical stability in terms of cardiovascular system [45, 46]. Thirdly, it has been demonstrated that not only cellular iron overload but also iron deficiency weakens the cellular defense against oxidative stress and abilities to adopt to energetic debt [47••, 48].

A separate issue is the recently raised question about possible interactions between intravenous iron/FCM therapy and concomitant pharmacological sodium-glucose co-transporter type 2 (SGLT2) inhibition [49, 50]. Namely, according to the hypothesis of “cytosolic iron repletion,” SGLT2 inhibitors both stimulate the sirutin-1 signaling and suppress systemic inflammation, which two parallel mechanisms lead to increased erythropoiesis and iron utilization (by “releasing” it for metabolic needs through decreased hepcidin), respectively [49, 50]. Therefore, they are not without merit and require verification in the rigor of an RCT the questions, firstly, about the possible overlapping effects of increasing the cellular iron pool through the combined use of FCM and SGLT2 inhibitors, and secondly, about the possible excessive stimulation of erythropoiesis due [49, 50].

## Conclusions

Regarding iron supplementation in iron-deficient patients with HF, as of early 2023, we generally know who, when, and how to treat, but we still do not know what the exact mechanisms translate into clinical benefits, but the latter is already an exciting journey from everyday clinical practice into the field of translational molecular medicine.
